# How loneliness relates to health, wellbeing, quality of life, and healthcare resource utilisation and costs across multiple age groups in the UK

**DOI:** 10.1371/journal.pone.0327671

**Published:** 2025-09-03

**Authors:** Nia Morrish, Anne Spencer, Antonieta Medina-Lara

**Affiliations:** 1 Public Health Economics Group, Department of Public Health and Sport Sciences, University of Exeter, Faculty of Health and Life Sciences, St Luke’s Campus, Exeter, United Kingdom; 2 Health Economics Group, Department of Health and Community Sciences, University of Exeter, Faculty of Health and Life Sciences, St Luke’s Campus, Exeter, United Kingdom; Cardiff University, UNITED KINGDOM OF GREAT BRITAIN AND NORTHERN IRELAND

## Abstract

Increasing evidence of its detrimental impact has brought loneliness to the forefront of public health in recent years. Loneliness has been recognised as a cross-cutting theme for Healthy Ageing by the World Health Organisation and there is increasing need to better understand its wide-ranging health, wellbeing, and economic impacts across the wider population. This study utilises data from wave 13(2021–2023) of the Understanding Society UK Household Longitudinal Study to evaluate health and economic outcomes associated to loneliness (UCLA 3-item scale). Outcomes include the General Health Questionnaire, Short Form Health Survey, Short Warwick-Edinburgh Mental Well-being Scale, and costed GP, outpatient, and inpatient visits. Generalised Linear Modelling is applied to adjust for demographic characteristics, and subgroup analysis is conducted to consider costs in different age groups. Complete data provided observations for 23,071 respondents. Average marginal effects found loneliness is associated to higher mental distress, lower positive mental wellbeing, poorer physical and mental functioning, and higher healthcare service use. Overall, there is approximately a £900 cost difference in healthcare use between lonely and non-lonely respondents. Cost difference increases with age, and for more severe loneliness forms a U-shape with the greatest costs in younger and older adults. Additionally, difference in mean is only statistically significant across all models for 16- to 24-year-olds, suggesting importance in targeting young adult healthcare resource use. This is the first study to consider age-based analysis of health-related costs of loneliness in the UK. It also adds to the literature by considering validated wellbeing and health-related outcomes in a large UK based population. Findings motivate tackling young adult loneliness to support their health, wellbeing, quality of life, and potential overuse of healthcare services. This study also supports the pressing need for greater economic evaluation of loneliness and loneliness interventions.

## Background

Loneliness has been described as the next critical public health issue [1] with a detrimental impact on a range of cognitive, behavioural, health, and economic outcomes [2–6]. Loneliness is defined as a subjective experience encapsulating the deficit between one’s desired and actual social relationships [7]. Thus, an individual may be lonely without being socially isolated as it encapsulates quality alongside quantity of social interaction. Loneliness has been increasingly recognised as a ‘priority public health problem and policy issue’ by the World Health Organisation (WHO) [8]. The WHO has also recognised loneliness as a key theme crosscutting their objectives for the United Nations Decade of Healthy Ageing (2021–2030). This, and existing research, has particularly emphasised the importance of considering loneliness across all age groups due to its wide-ranging impacts [8,9], and the non-linear relationship with age, observing peaks in early, mid, and older age [10,11]. Additionally, national Governments, such as the UK, have recently increased their consideration of loneliness in policymaking, and prioritised improvement of the evidence base [12].

There is increasing evidence of the detrimental impact of loneliness on health with loneliness linked to poor health conditions including all-cause mortality, cardiovascular disease, and mental health [13–15]. Research also suggests that loneliness is associated to poorer quality of life [16–19]. Though the definition of quality of life is diverse, it is a subjective measure of an individual’s well-being [20] that includes but is not limited to physical, social, spiritual, and emotional well-being. The WHO additionally notes that quality of life is influenced by an individual’s perception of their position in the context of culture, expectations, standards, and concerns [21]. Existing research found the relationship between loneliness and lower quality of life to be particularly strong when considering emotional aspects of loneliness and mental health [22]. This existing study is however restricted to an older adult population and encourages greater consideration of socio-demographic characteristics suggesting benefit to consideration of age-related differences in the evaluation of loneliness and quality of life. Additionally, research in an older adult population with coronary heart disease found baseline loneliness to predict physical and mental quality of life, remaining significant when adjusting for depression [23] which can be a predictor of loneliness [18]. Research to date considering the relationship between loneliness and quality of life focuses on older adults, with fewer studies [24] and more limited understanding of the association in the wider population [24,25]. Meanwhile, research on the prevalence of loneliness has identified a non-linear association with age [10,11] reinforcing the need to look at its associations across the life course. This is further reinforced by indications that factors related to loneliness can be understood from an age-normative perspective [26] with, for example, perceived health [26], and poor health and functional limitations [27], associating to loneliness with different magnitudes at different ages.

There is also some evidence that loneliness can lead to overuse of healthcare services acting as a predictor of re-hospitalisation [28], greater number of emergency department visits [13], more frequent GP visits [25,29], and institutionalisation [30]. While these associations between loneliness and healthcare resource use have been identified, quantification of the excess cost associated to service use is limited. A recent review on the economic costs of loneliness recognises literature in this area to be scarce and highlights the need for more research into the burden of loneliness, and potential benefits of intervention for younger people [25]. Particular evidence gaps in this research area arise around excess healthcare costs, economic burden, and cost-effectiveness of loneliness interventions [25,31]. This accentuates a lack of understanding of the association between loneliness and health-related costs in the general population. Research conducted in the Netherlands [31,32] suggests that loneliness slightly increases GP contacts, emergency room visits, and hospital admission days [32]. These findings from the Netherlands could be influenced by the presence of out-of-pocket healthcare expenditure within the basic insurance plan, which could impact healthcare utilisation. There is therefore benefit to considering the impact of loneliness on healthcare resource utilisation in countries where healthcare is free at the point of need, as with the National Health Service in the UK, where the financial pressures associated to healthcare usage are lessened. This is particularly of interest given some evidence of loneliness relating to fewer dentist visits when the individual is also under financial strain [28].

Overall, the impact of loneliness is wide ranging and multifactorial. To date, research focuses on older adults with limited understanding for the wider population or healthcare service use. This study therefore looks to quantify health and economic outcomes associated to loneliness in the UK general population. Using the Understanding Society dataset, it first considers health, wellbeing, and quality of life measures to understand the association of loneliness with these outcomes. Second, it estimates the costs associated with healthcare resource use across individuals experiencing different levels of loneliness across different age groups. By linking these two objectives, this study deepens understanding of how loneliness affects individual’s health, wellbeing and quality of life, while also quantifying its wider impact on the public healthcare system through differences in resource use. It also motivates the pressing need for cost-effective interventions to improve loneliness in the UK population.

## Methods

### Data

Data came from the Understanding Society UK Household Longitudinal Study [33], a longitudinal survey containing approximately 40,000 households. Understanding Society collects data from UK adults aged 16 and over through self-completion online surveys or through face-to-face interviews. These data are collected with the aim of studying the effect of social, economic, and policy change on wellbeing in the UK. Data are captured for all key variables of interest for this study, being loneliness, wellbeing, quality of life, and healthcare resource use, using validated scales where available. Loneliness has been measured in Understanding Society from wave 9 (2017−2019) onwards. This research utilises data from wave 13 (2021−2023) of the study, the most recent data available at the time of analysis with a post-COVID-19 focus. This avoids potential confounding from the pandemic in earlier waves (waves 10–12) and provides insight on the relationships in the post-pandemic ‘new normal’ [34]. Data are included from individuals with complete responses for all the variables of interest to this study.

### Demographics

Demographic variables for age, sex, ethnicity, marital status, education, and employment status are reported. Age is reported as a continuous variable while sex (female/male), ethnicity (white/non-white), marital status (married/not married), education (higher/other education), and employment (employed/unemployed) are transformed and reported as binary.

### Loneliness measures

Loneliness was assessed in two ways; using a single item question, and using the UCLA 3-item scale [35]. The single item question directly asks, ‘How often do you feel lonely?’ and has three response options: 1) hardly ever or never, 2) some of the time, 3) often. The UCLA 3-item scale consists of three questions each indirectly related to loneliness: ‘how often do you feel you lack companionship?’, ‘how often do you feel left out?’, and ‘how often do you feel isolated from others?’. Each of the three UCLA questions also have three response options: 1) hardly ever or never, 2) some of the time, 3) often. Thus, a higher score indicates a greater level of loneliness. For the UCLA 3-item scale the loneliness score is calculated as the sum of all items yielding a scale from three to nine. However, because the scale is constructed from categorical responses rather than continuous data, we interpret the UCLA loneliness score using the initial categories 1) UCLA loneliness score = 3–4 as ‘hardly ever or never’, 2) UCLA loneliness score = 5–7 as ‘some of the time’, 3) UCLA loneliness score = 8–9 as ‘often’ lonely [36]. This enables the analysis to retain the nuance inherent in the subjective nature of loneliness, recognising its varying levels of severity, commonly described as ‘some of the time’ or ‘often’, and is consistent with existing research which recognises the importance of the categorical classification [37]. Furthermore, this classification aligns with the format of the single item question of loneliness, allowing for consistency across instruments.

In our analysis we use these categorical definitions of loneliness to transform the responses to the UCLA score, and to the single item question, into binary variables. For primary analysis, we distinguish respondents that report being lonely ‘often’ and contrast these with respondents who report being lonely either ‘some of the time’ or ‘hardly ever or never’. This allows us to evidence the effect of experiencing more severe loneliness. For secondary analysis we seek to understand the impact of having any experience of loneliness and so group together respondents that report feeling lonely ‘some of the time’ or ‘often’, and contrast with respondents who report being ‘hardly ever or never’ lonely. This approach is consistent with the classifications used in existing research [3]. It aids interpretation of the loneliness score and facilitates consideration of any differences based on severity of experience.

### Quality of life (QoL) measures

QoL measures were selected to cover various facets of quality of life and wellbeing. Measures were included for physical and mental health (12 item Short Form Health Survey, SF-12), psychological distress (12 item General Health Questionnaire, GHQ-12), and wellbeing (Short Warwick-Edinburgh Mental Well-being Scale, SWEMWBS).

#### SF-12.

The SF-12 covers 12 domains (physical function, role-physical, bodily pain, general health, vitality, social function, role-emotional, mental health) with a recall period of 4-weeks [38]. Domains can be summarised into two summary scores, the Physical Health Component (PCS) and Mental Health Component (MCS). Component scores range from 0 (low functioning) to 100 (high functioning). A score of 50 or less on the PCS signifies a physical condition, and a score of 42 or less on the MCS indicates clinical depression [39,40].

#### GHQ-12.

The 12-item version of the GHQ-12 was designed as a measure of psychological distress [41,42]. The questionnaire covers 12 domains (concentration, loss of sleep, playing a useful role, capability in decision making, feeling under strain, problems overcoming difficulties, enjoying day-to-day activities, ability to face problems, feeling unhappy or depressed, losing confidence, thinking of yourself as worthless, and general happiness), using a recall period of ‘the last few weeks’. Responses differ depending on whether the question is positively or negatively framed though are consistent in ranging on a scale from 1 (better health) to 4 (poorer health/greater mental distress). Valid answers are recoded onto a single scale using either a Likert or Caseness method. The Likert method gives a total score ranging from 0 (least distressed) to 36 (most distressed), while the Caseness method runs on a scale from 0 (least distressed) to 12 (most distressed) with a score greater than four indicating poor mental health [43,44].

#### SWEMWBS.

The SWEMWBS [45] measures mental wellbeing with seven positively worded statements about thoughts and functioning using a recall period of two weeks. Five response categories are available ranging from ‘none of the time’ to ‘all of the time’. Scores are summed from each of the seven items to create a total score ranging from 7 to 35 with higher scores indicating higher positive mental wellbeing. A score of less than 19 is indicative of possible clinical depression, score between 18 and 20 of possible mild depression, and a score of 29 and above suggests high mental wellbeing [46].

### Healthcare resource use and costs

Participants were asked about the number of GP appointments, outpatient and inpatients visits over a 12-month period. These services were selected given evidence of their potential association to loneliness in other settings [25,29,31,32]. To determine the treatment costs, unit costs were assigned to the number of healthcare encounters to understand the costs to the NHS associated to loneliness. NHS costs were estimated using unit costs from the 2023 Personal Social Services Research Unit [47]. As detailed in S1 Table, a GP visit (10-minutes consultation at surgery) would cost £49, an outpatient visit cost £217, and an inpatient day case costs £1,111 per day.

Costs were combined with mean resource use for analysis using standard methodology for economic evaluations [48,49]. Inpatient number of days were reported as a continuous free-text response, however, number of GP appointments and outpatient visits were reported on a predefined scale with response options grouped into ranges. Thus, GP and outpatient costs were generated for lower and upper bound estimates. Some participants reported to have attended more than 10 GP appointments or outpatient visits. In these cases, a lower bound of 11 visits was assigned, while the upper bound was based on existing literature. For GP visits an upper bound of 25 visits was assigned as identified in recent research on frequent attenders [50]. For outpatient visits data were taken from the World Health Organisation outpatient contacts per person per year [51] for the UK yielding an upper bound of 13 outpatient visits per year.

### Statistical and econometric analysis

Descriptive statistics reporting mean, median, standard deviation and range are used to describe the data. Median costs are reported given the skewness of cost data. Unadjusted difference in mean QoL measures, and healthcare resource use outcomes for those reporting feeling lonely versus not lonely are reported to indicate how loneliness interacts with physical and mental health, psychological distress, well-being, behavioural attitudes and its costs to the NHS. Findings were then adjusted for age, sex, ethnicity, marital status, level of education and employment using a generalised linear model (GLM) with gamma distribution and log link [52]. Models included loneliness as a binary independent variable and each of the QoL and healthcare resource use outcomes as dependent variables in turn.

The UCLA score is used in the main analysis with the direct loneliness question included as sensitivity analysis to allow comparison between loneliness measures. Primary analysis defined loneliness as the experience of feeling lonely ‘often’. Secondary analysis expanded this definition to consider an individual as lonely when experiencing loneliness often or sometimes.

Adjusted and unadjusted models were run with dependent variables for upper and lower bound healthcare service use, and related cost estimates. Total costs were then estimated across six age groups [53] to consider changes across the life course. Analyses are conducted using Stata/SE version 18. Missing responses were excluded from analysis.

### Ethical approval statement

This study has been approved by the University of Exeter Faculty of Health and Life Sciences Sports and Health Sciences Ethics Committee. The University of Essex Ethics Committee has approved all data collection on Understanding Society main study, COVID-19 surveys and innovation panel waves, including asking consent for all data linkages except to health records. Requesting consent for health record linkage was approved at Wave 1 by the National Research Ethics Service (NRES) Oxfordshire REC A (08/H0604/124), at BHPS Wave 18 by the NRES Royal Free Hospital & Medical School (08/H0720/60) and at Wave 4 by NRES Southampton REC A (11/SC/0274).

## Results

### Descriptive statistics

Descriptive statistics for the sample are reported in Table 1. Complete data for all included measures provided observations for 23,071 respondents. Respondents had a mean age of 50 (SD = 19, range 16–101), and were 56% (12,800/23,071) female. Most of the sample were of white ethnicity (84%; 19,342/23,071). The sample was quite evenly split between married (54%; 12,343/23,071) and not currently married (47%; 10,728/23,071), and between having received higher education (43%; 9,976/23,071) or not (57%; 13,005/23,071). Two thirds of the respondents were currently employed or in education (65%; 14,928/23,071). For both the UCLA loneliness scale and the direct loneliness score, around 60% of respondents reported feeling lonely ‘hardly ever or never’, around 32% felt lonely some of the time, and around 8% indicated they felt lonely ‘often’. There is slight variation in these proportions for each of the UCLA dimensions as for example 8% of respondents reported ‘often’ feeling ‘lack of companionship’, ‘isolated from others’, while slightly fewer (6%) said they ‘often’ feel ‘left out’.

**Table 1:** Descriptive statistics, n(%).

Demographics	
N	23,071
Age, *mean(SD)[range]*	49.750(18.527) [16,101]
Sex	
Female[0]	12,800(55.5%)
Male [1]	10,271(44.5%)
Ethnicity	
White[0]	19,342(83.8%)
Non-white [1]	3,729(16.2%)
Marital status	
Married or civil partnership[0]	12,343(53.5%)
Not currently married or in civil partnership [1]	10,728(46.5%)
Education	
Higher education[0]	9,976(43.2%)
Other education [1]	13,095(56.8%)
Employment	
Employed or in education[0]	14,928(64.7%)
Unemployed or other employment status [1]	8,143(35.3%)
**Loneliness**	
How often feels lack of companionship*	
Hardly ever or never [1]	13,153(57.0%)
Some of the time [2]	7,994(34.6%)
Often [3]	1,924(8.3%)
How often feels left out*	
Hardly ever or never [1]	14,300(62.0%)
Some of the time [2]	7,412(32.1%)
Often [3]	1,359(5.9%)
How often feels isolated from others*	
Hardly ever or never [1]	13,540(58.7%)
Some of the time [2]	7,646(33.1%)
Often [3]	1,885(8.2%)
How often feels lonely	
Hardly ever or never [1]	13,819(59.9%)
Some of the time [2]	7,354(31.9%)
Often [3]	1,898(8.2%)
**Wellbeing and quality of life**	**mean(SD)[range]**
GHQ-12 (Likert:0-1-2-3)	11.716(5.733)[0,36]
GHQ-12 (Caseness:0-0-1-1)	1.947(3.214)[0,12]
SF12-PCS	49.797(10.505)[5.9,74.17]
SF12-MCS	47.449(10.979)[0,74.46]
SWEMWBS	24.336(4.842) [7,35]
**Health care service use**	**N(%)**
Visited GP in last 12 months	
0[0]	9,464(41.0%)
1-2 [1]	8,696(37.7%)
3-5 [2]	3,322(14.4%)
6-10 [3]	1,054(4.6%)
>10 [4]	535(2.3%)
Hospital or clinic out-patient last 12 months	
0[0]	14,860(64.4%)
1-2 [1]	5,581(24.2%)
3-5 [2]	1,697(7.4%)
6-10 [3]	568(2.5%)
>10 [4]	365(1.6%)
Hospital or clinic in-patient last 12 months	
No[0]	21,603(93.6%)
Yes [1]	1,468(6.4%)
In-patient number of days, *mean(SD)[range]*	0.505(4.443)[0,200]

*Used to create UCLA loneliness score.

[]=data code.

The mean SF-12 PCS score for the sample was 49.8 (50 or less signifies a physical condition), and mean SF-12 MCS score was 47.4 (42 or less indicates clinical depression). The GHQ-12 mean score of 11.7 using the Likert scale, and 1.9 using the Caseness coding with both numbers indicating low mental distress. Mean SWEMWBS score for the sample was 24.3, similar to the population norm reported in previous studies [54], and suggesting middling wellbeing.

Most of the sample visited a GP no more than two times in the last 12 months (79%), of which 41% did not visit a GP at all in the last year. Meanwhile 2% of respondents visited a GP more than ten times in 12 months. Two thirds (64%) of the sample did not attend a hospital or clinic out-patient in the last 12 months while 2% attended more than 10 times. Most respondents (94%) had not attended a hospital or clinic as an in-patient in the last 12 months, 6% of the sample did attend, thus overall, the full sample reported a mean of 0.5 (SD = 4.4, range 0–200) inpatient days.

### Difference in mean

Across all variables a greater association is observed between loneliness and the QoL related outcomes when defining loneliness as experiencing lonely often, than when including any experience of loneliness (often or sometimes) in the analysis. The average marginal effect (predicted mean difference) is slightly smaller for the adjusted than the unadjusted model, as would be expected when accounting for covariates. In both models however, for each variable a statistically significant (p < 0.05) average marginal effect is observed. Unadjusted scores for both loneliness measures are reported in S2 Table.

The main difference in mean analysis reported in this section is based on the UCLA loneliness scale, as reported in Table 2. The sensitivity analysis considering the single item question for loneliness is also reported in Table 2. There is little difference in key results when comparing the UCLA scale to the single item question for loneliness, though statistical significance of the difference between loneliness measures was not explored in this study.

**Table 2:** Difference in mean (adjusted average marginal effects)ᶲ^Ϯ.^

	UCLA						Direct question					
	Lonely = often lonely			Lonely = sometimes or often lonely			Lonely = often lonely			Lonely = sometimes or often lonely		
	Lonely N = 1,385	Not lonely N = 21,686	Difference N = 23,071	Lonely N = 9,475	Not lonely N = 13,596	Difference N = 23,071	Lonely N = 1,898	Not lonely N = 21,173	Difference N = 23,071	Lonely N = 9,252	Not lonely N = 13,819	Difference N = 23,071
**Wellbeing and Quality of Life**												
SF12-PCS	46.72 (0.25) [46.22,47.22]	50.00 (0.07) [49.86,50.13]	−3.28*** (0.26) [−3.79,-2.76]	48.27 (0.10) [48.07,48.46]	50.88 (0.09) [50.71,51.05]	−2.61*** (0.14) [−2.88,-2.35]	46.96 (0.22) [46.53,47.38]	50.06 (0.07) [49.92,50.19]	−3.10*** (0.23) [−3.55,-2.65]	48.33 (0.10) [48.13,48.53]	50.80 (0.09) [50.63,50.97]	−2.48*** (0.14) [−2.75,-2.21]
SF12-MCS	33.56 (0.20) [33.17,33.95]	48.31 (0.07) [48.17,48.45]	−14.75*** (0.21) [−15.17,-14.34]	41.56 (0.09) [41.38,41.74]	51.49 (0.09) [51.30,51.67]	−9.92*** (0.13) [−10.18,-9.66]	33.94 (0.17) [33.60,34.27]	48.63 (0.07) [48.49,48.77]	−14.69*** (0.19) [−15.05,-14.32]	41.41 (0.09) [41.23,41.59]	51.42 (0.09) [51.24,51.60]	−10.01*** (0.13) [−10.27,-9.75]
GHQ-12 (Likert)	19.26 (0.23) [18.80,19.72]	11.22 (0.03) [11.15,11.28]	8.04*** (0.24) [7.58,8.51]	14.69 (0.06) [14.56,14.81]	9.61 (0.03) [9.54,9.68]	5.08*** (0.07) [4.94,5.22]	19.10 (0.20) [18.72,19.48]	11.03 (0.03) [10.97,11.10]	8.07*** (0.20) [7.68,8.46]	14.72 (0.07) [14.59,14.85]	9.67 (0.04) [9.60,9.74]	5.05*** (0.08) [4.91,5.20]
GHQ-12 (Caseness)	5.78 (0.27) [5.24,6.31]	1.68 (0.02) [1.64,1.72]	4.10*** (0.28) [3.56,4.64]	3.35 (0.07) [3.22,3.49]	0.91 (0.02) [0.88,0.94]	2.45*** (0.07) [2.31,2.58]	5.72 (0.24) [5.26,6.18]	1.57 (0.02) [1.53,1.61]	4.15*** (0.24) [3.68,4.61]	3.35 (0.07) [3.21,3.48]	0.95 (0.02) [0.92,0.98]	2.40*** (0.07) [2.26,2.53]
SWEMWBS	18.32 (0.09) [18.14,18.51]	24.71 (0.03) [24.65,24.77]	−6.39*** (0.10) [−6.58,-6.20]	21.56 (0.04) [21.49,21.64]	26.25 (0.04) [26.17,26.33]	−4.69*** (0.06) [−4.80,-4.58]	18.69 (0.08) [18.54,18.85]	24.83 (0.03) [24.77,24.89]	−6.14*** (0.09) [−6.31,-5.97]	21.55 (0.04) [21.47,21.62]	26.19 (0.04) [26.11,26.27]	−4.64*** (0.06) [−4.76,-4.53]
**Health care service use (last 12 months)**												
GP visit	1.21 (0.04) [1.14,1.28]	0.88 (0.01) [0.86,0.89]	0.33*** (0.04) [0.26,0.40]	1.03 (0.01) [1.01,1.05]	0.80 (0.01) [0.79,0.82]	0.23*** (0.01) [0.20,0.26]	1.22 (0.03) [1.16,1.28]	0.87 (0.01) [0.85,0.88]	0.35*** (0.03) [0.29,0.41]	1.03 (0.01) [1.01,1.06]	0.80 (0.01) [0.79,0.82]	0.23*** (0.01) [0.20,0.26]
Hospital or clinic out-patient	0.70 (0.03) [0.64,0.77]	0.52 (0.01) [0.51,0.53]	0.19*** (0.03) [0.12,0.25]	0.61 (0.01) [0.59,0.63]	0.47 (0.01) [0.46,0.49]	0.14*** (0.01) [0.11,0.16]	0.71 (0.03) [0.66.0.77]	0.51 (0.01) [0.50,0.53]	0.20*** (0.03) [0.15,0.26]	0.62 (0.01) [0.60,0.64]	0.47 (0.01) [0.46,0.48]	0.15*** (0.01) [0.12,0.18]
Hospital or clinic in-patient	0.10 (0.01) [0.08,0.13]	0.06 (0.00) [0.06,0.07]	0.04*** (0.01) [0.02,0.06]	0.08 (0.00) [0.07,0.08]	0.06 (0.00) [0.05,0.06]	0.02*** (0.00) [0.01,0.03]	0.10 (0.01) [0.08,0.12]	0.06 (0.00) [0.06,0.07]	0.04*** (0.01) [0.02,0.06]	0.08 (0.00) [0.07,0.08]	0.06 (0.00) [0.05,0.06]	0.02*** (0.00) [0.01,0.03]
In-patient number of days	1.10 (0.25) [0.61,1.58]	0.46 (0.03) [0.40,0.53]	0.64** (0.25) [0.15,1.12]	0.64 (0.06) [0.52,0.76]	0.40 (0.04) [0.33,0.47]	0.24*** (0.06) [0.11,0.36]	0.99 (0.20) [0.60,1.37]	0.46 (0.03) [0.40,0.53]	0.53** (0.19) [0.14,0.91]	0.65 (0.06) [0.53,0.77]	0.40 (0.03) [0.33,0.47]	0.25*** (0.06) [0.12,0.38]

^ᶲ^Mean(SE)[95%CI].

^Ϯ^Adjusted using GLM with gamma distribution and log link. Covariates: age, sex, ethnicity, marital status, education, employment status.

*p < 0.05,**p < 0.01,***p < 0.001.

### Psychological distress, physical and mental health and wellbeing

The physical subscale of the SF-12 found lower functioning in individuals experiencing loneliness than no loneliness with adjusted average marginal effect (AME) −3.28 reducing to −2.61 when including sometimes lonely in the definition for loneliness. For the mental component score adjusted difference in mean determined that the AME fell between −9.92 (lonely often) and −14.75 (often or sometimes lonely) indicating lower mental functioning in those experiencing loneliness.

For the SF-12, a score of 50 or less on the PCS signifies a physical condition, and a score of 42 or less on the MCS indicates clinical depression [39,40]. For the ‘not lonely’ group, the mean (adjusted) SF-12 component scores for both the physical and mental subscales fall outside these thresholds, however for those experiencing loneliness, mean physical (PCS) and mental (MCS) scores indicate the presence of a physical condition, and clinical depression, respectively. Findings indicate loneliness is associated to greater psychological distress as measured by the GHQ-12 scale. Using the GHQ-12 Likert scale adjusted AME was 8.04 for those experiencing loneliness often and 5.08 when including feeling lonely some of the time in the definition for loneliness. For the Caseness scale difference in means were 4.10 and 2.45 respectively. Adjusted mean scores for the Caseness scale indicate that when loneliness is more extreme (lonely defined as experiencing loneliness often) individuals experiencing loneliness also experience poor mental health (GHQ-12 score more than 4), whereas the threshold is not reached for individuals not experiencing loneliness. The threshold is not reached for either group when including individuals experiencing loneliness ‘some of the time’ in the definition for loneliness.

Finally, lower positive mental wellbeing (SWEMWBS) with adjusted AME −6.39 (lonely often) and −4.69 (lonely often or sometimes) is observed. Again, the broader definition for loneliness indicates a score above the threshold for depression for both lonely and non-lonely individuals. However, when restricting loneliness to something experienced ‘often’, individuals who experience loneliness are also seen to experience mild depression with a SWEMWBS score in the threshold 18−20. Each of these results, for the SF-12, GHQ-12, and SWEMWBS achieve statistical significance of p < 0.001.

### NHS service use and related costs

Table 2 shows that individuals experiencing loneliness have more GP appointments and more out-patient visits over a 12-month period than individuals not experiencing loneliness. For inpatient visits, AME for whether an inpatient visit occurred or not (binary variable) was twice as large when restricting the definition of loneliness to ‘often’ only (AME = 0.02 compared to 0.04). Additional detail on the number of in-patient days is also provided with adjusted AME 0.64 (often) and 0.24 for the wider definition of loneliness (often or sometimes) indicating a higher number of inpatient days associated to individuals experiencing loneliness than not experiencing loneliness. Median scores (unadjusted) are also reported for all variables, however no difference was observed between groups, as reported in S2 Table.

Table 3 reports the adjusted AME for GP and outpatient visits, based on the lower and upper bounds of the response options. The adjusted AME for GP visits is estimated to be between 0.74 and 1.47 where loneliness is defined as something experienced ‘often’, and between 0.48 and 0.93 when widening the definition of loneliness to include those experiencing it ‘some of the time’. In all cases more GP visits were observed amongst those experiencing loneliness. For outpatient hospital or clinic visits the adjusted difference in mean is estimated between 0.37 and 0.53 (often) or between 0.25 and 0.38 (often or sometimes) with individuals identified as lonely attending more frequently. In each case, for GP and for outpatient visits, the higher estimates are derived from the upper bound. Unadjusted median service use is reported in S3 Table with the same median use of GP and outpatient services reported in each group.

**Table 3:** Adjusted cost of health care service use based on mean service use ᶲ^Ϯ.^

Health care service (use in last 12 months)	Unit cost	UCLA						Direct question					
		Lonely=often lonely			Lonely=sometimes or often lonely			Lonely=often lonely			Lonely=sometimes or often lonely		
		Lonely N=1,385	Not lonely N=21,686	Difference N=23,071	Lonely N=9,475	Not lonely N=13,596	Difference N=23,071	Lonely N=1,898	Not lonely N=21,173	Difference N=23,071	Lonely N=9,252	Not lonely N=13,819	Difference N=23,071
Mean service use (adjusted)													
GP visit [lower bound]	n/a	2.04 (0.09) [1.87,2.20]	1.29 (0.01) [1.26,1.32]	0.74*** (0.09) [0.57,0.91]	1.62 (0.03) [1.57,1.67]	1.14 (0.02) [1.11,1.17]	0.48*** (0.03) [0.42,0.54]	2.04 (0.07) [1.90,2.19]	1.27 (0.01) [1.25,1.30]	0.77*** (0.08) [0.62,0.92]	1.62 (0.03) [1.57,1.68]	1.14 (0.02) [1.11,1.18]	0.48*** (0.03) [0.42,0.54]
GP visit [upper bound]	n/a	3.89 (0.18) [3.55,4.24]	2.42 (0.03) [2.36,2.47]	1.47*** (0.18) [1.12,1.82]	3.05 (0.05) [2.95,3.16]	2.12 (0.03) [2.06,2.18]	0.93*** (0.06) [0.81,1.05]	3.90 (0.15) [3.60,4.19]	2.38 (0.03) [2.32,2.44]	1.52*** (0.15) [1.21,1.82]	3.06 (0.05) [2.95,3.16]	2.14 (0.03) [2.07,2.20]	0.92*** (0.06) [0.80,1.04]
Hospital or clinic out-patient visit [lower bound]	n/a	1.14 (0.07) [1.00,1.28]	0.77 (0.01) [0.74,0.79]	0.37*** (0.07) [0.23,0.51]	0.94 (0.02) [0.89,0.99]	0.69 (0.01) [0.66,0.72]	0.25*** (0.03) [0.20,0.30]	1.16 (0.06) [1.03,1.28]	0.76 (0.01) [0.73,0.78]	0.40*** (0.06) [0.27,0.53]	0.96 (0.02) [0.91,1.01]	0.68 (0.01) [0.65,0.71]	0.28*** (0.03) [0.23,0.34]
Hospital or clinic out-patient visit [upper bound]	n/a	1.81 (0.10) [1.62,2.00]	1.28 (0.02) [1.24,1.32]	0.53*** (0.10) [0.34,0.73]	1.54 (0.03) [1.47,1.60]	1.16 (0.02) [1.12,1.20]	0.38*** (0.04) [0.31,0.46]	1.85 (0.09) [1.68,2.01]	1.27 (0.02) [1.23,1.30]	0.58*** (0.09) [0.41,0.75]	1.57 (0.03) [1.50,1.64]	1.14 (0.02) [1.10,1.18]	0.43*** (0.04) [0.35,0.50]
Hospital or clinic in-patient (number of days)	n/a	1.10 (0.25) [0.61,1.58]	0.46 (0.03) [0.40,0.53]	0.64** (0.25) [0.15,1.12]	0.64 (0.06) [0.52,0.76]	0.40 (0.04) [0.33 0.47]	0.24*** (0.06) [0.11,0.36]	0.99 (0.20) [0.60,1.37]	0.46 (0.03) [0.40,0.53]	0.53** (0.19) [0.14,0.91]	0.65 (0.06) [0.53, 0.77]	0.40 (0.03) [0.33,0.47]	0.25*** (0.06) [0.12,0.38]
Cost of mean service use (£)													
GP visit [lower bound]	£49	99.78 (4.21) [91.53, 108.04]	63.34 (0.70) [61.97, 64.70]	36.45*** (4.27) [28.07,44.83]	79.32 (1.29) [76.80, 81.84]	55.81 (0.77) [54.29, 57.32]	23.52*** (1.50) [20.58,26.45]	100.09 (3.63) [92.98, 107.21]	62.38 (0.70) [61.01, 63.74]	37.72*** (3.70) [30.46,44.97]	79.52 (1.32) [76.94, 82.11]	56.08 (0.78) [54.56, 57.60]	23.44*** (1.53) [20.43,26.44]
GP visit [upper bound]	£49	190.66 (8.61) [173.80, 207.53]	118.50 (1.40) [115.76, 121.24]	72.16*** (8.73) [55.05,89.28]	149.54 (2.58) [144.48, 154.60]	104.00 (1.54) [100.99, 107.01]	45.54*** (3.00) [39.67,51.42]	190.88 (7.40) [176.37, 205.38]	116.61 (1.39) [113.89, 119.34]	74.26*** (7.55) [59.47,89.06]	149.77 (2.64) [144.59, 154.95]	104.62 (1.54) [101.59, 107.65]	45.15*** (3.07) [39.1351.17]
Hospital or clinic out-patient visit [lower bound]	£217	246.43 (15.55) [215.95, 276.91]	166.76 (2.84) [161.20, 172.32]	79.67*** (15.73) [48.84,110.49]	203.90 (5.11) [193.89, 213.92]	149.41 (3.15) [143.25, 155.58]	54.49*** (5.84) [43.04,65.94]	251.31 (13.66) [224.53, 278.08]	164.49 (2.83) [158.94, 170.03]	86.82*** (13.86) [59.66,113.98]	208.89 (5.35) [198.40, 219.37]	147.37 (3.09) [141.32, 153.41]	61.52*** (6.04) [49.68,73.36]
Hospital or clinic out-patient visit [upper bound]	£217	392.98 (21.28) [351.28, 434.69]	277.88 (4.02) [270.00,285.75]	115.11*** (21.55) [72.87,157.35]	333.81 (7.15) [319.80, 347.82]	251.15 (4.51) [242.31, 259.99]	82.66*** (8.27) [66.44,98.87]	400.61 (18.70) [363.97, 437.26]	274.54 (4.01) [266.68, 282.41]	126.07*** (19.01) [88.82,163.32]	341.06 (7.47) [326.41, 355.70]	248.40 (4.43) [239.72, 257.09]	92.65*** (8.53) [75.92,109.38]
Hospital or clinic in-patient (number of days)	£1,111	1220.21 (274.82) [681.57, 1758.84]	513.98 (36.77) [441.92, 586.03]	706.23** (274.76) [167.72,1244.74]	709.09 (67.12) [577.53, 840.64]	447.08 (39.35) [369.95, 524.21]	262.01*** (72.20) [120.49,403.52]	1097.32 (217.11) [671.79, 1522.86]	512.60 (37.43) [439.24, 585.97]	584.72** (216.50) [160.38,1009.06]	721.79 (68.67) [587.20, 856.38]	445.51 (38.34) [370.36, 520.66]	276.28*** (72.05) [135.06,417.50]
**Total health service cost** *[lower bound]*	n/a	1557.37 (229.03) [1108.49, 2006.26]	744.02 (32.59) [680.13, 807.90]	813.35*** (230.00) [362.57,1264.14]	999.04 (60.24) [880.98, 1117.10]	646.65 (35.19) [577.68, 715.62]	352.39*** (66.22) [222.60,482.18]	1429.52 (183.93) [1069.02, 1790.02]	738.85 (33.09) [674.00, 803.70]	690.67*** (184.83) [328.41,1052.93]	1019.17 (62.15) [897.36, 1140.98]	643.46 (34.29) [576.26, 710.66]	375.71*** (66.97) [244.44,506.97]
**Total health service cost** *[upper bound]*	n/a	1796.44 (214.52) [1375.99, 2216.88]	910.95 (31.54) [849.13, 972.76]	885.49*** (215.84) [462.45,1308.53]	1204.14 (58.33) [1089.81, 1318.47]	794.72 (34.29) [727.52, 861.92]	409.42*** (64.85) [282.32,536.53]	1667.30 (174.00) [1326.27,2008.33]	903.63 (31.99) [840.93, 966.32]	763.67*** (175.40) [419.91,1107.44]	1226.32 (60.15) [1108.42, 1344.22]	791.64 (33.48) [726.03, 857.25]	434.68*** (65.72) [305.86,563.49]

^ᶲ^Mean(SE)[95%CI].

^Ϯ^Adjusted using GLM with gamma distribution and log link. Covariates: age, sex, ethnicity, marital status, education, employment status.

*p < 0.05,**p < 0.01,***p < 0.001.

Total NHS service costs combine GP, outpatient, and inpatient visits and is consistently higher for individuals experiencing loneliness compared to individuals not experiencing loneliness (Table 3). The adjusted difference in mean cost is higher when defining loneliness as feeling lonely ‘often’ with difference in cost expected between £813.35 and £885.49 per person, whereas when defining loneliness as feeling loneliness ‘often or sometimes’ service cost is expected to be between £352.39 and £409.42. Median unadjusted costs are reported in S3 Table. When using the wider definition of loneliness median service use cost is the same in each group. When restricting the definition of loneliness to be only those experiencing it often, a greater median cost is observed amongst those experiencing loneliness for both the lower (£147 vs £49) and upper bound (£245 vs £98). Considering the cost components (Table 3), the greatest adjusted difference in mean cost arises from inpatient days with AME £706.23 and £262.01 for the restricted (often) and less restricted (often or sometimes) definitions of loneliness respectively. In both cases higher costs arise in the ‘lonely’ group.

When defining loneliness as something experienced sometimes or often, age group analysis reveals the difference in cost between lonely and non-lonely groups increases in magnitude and statistical significance as individuals get older (Table 4). The difference in cost is larger in magnitude for all age groups other than over 75 years when restricting the definition of loneliness to something experienced ‘often’, i.e., a more severe experience. Additionally, individuals aged 16–24 years have higher health related costs than those aged 25–49, suggesting a U-shaped health-related cost of loneliness by age. Furthermore, difference in mean cost is only statistically significant for the upper and lower bound at age 16–24.

**Table 4:** Difference in health care service cost by ageᶲ^Ϯ.^

Age group	N	Difference in cost			
		Lonely = often lonely		Lonely = sometimes or often lonely	
		Lower bound	Upper bound	Lower bound	Upper bound
16-24	2,712	£423.50** (158.44) [112.96,734.03]	£473.18*** (148.30) [182.52,763.84]	£126.42* (58.60) [11.56,241.27]	£167.17** (61.30) [47.02,287.33]
25-34	3,039	£175.00 (201.91) [-220.74,570.73]	£245.81 (203.56) [-153.15,644.77]	£141.54 (106.21) [-66.64,349.72]	£177.97 (103.42) [-24.72,380.67]
35-49	5,237	£365.76 (240.42) [-105.45,836.97]	£433.84 (238.56) [-33.73,901.42]	£202.43* (88.00) [29.96,374.90]	£255.46** (89.48) [80.08,430.84]
50-64	6,446	£668.75 (377.45) [-71.04,1408.54]	£725.28* (359.07) [21.51,1429.05]	£402.14** (131.55) [144.32,659.97]	£457.82*** (130.40) [202.24,713.41]
65-74	3,440	£1959.56 (1020.65) [-40.87,3960.02]	£2063.78* (947.12) [207.46,3920.10]	£807.76** (272.99) [272.72,1342.81]	£854.49*** (261.03) [342.89,1366.09]
75+	2,197	£1129.57 (1196.18) [-1214.89,3474.03]	£1169.45 (1169.80) [-1123.32,3462.21]	£1549.14*** (482.70) [603.07,2495.21]	£1605.79*** (473.70) [677.36,2534.23]

^ᶲ^Mean(SE)[95%CI].

^Ϯ^Adjusted using GLM with gamma distribution and log link. Covariates: age, sex, ethnicity, marital status, education, employment status.

*p < 0.05,**p < 0.01,***p < 0.001

## Discussion

This study explores quality of life, wellbeing outcomes, and the costs to the NHS associated to loneliness in the UK general population. The average age of this sample is 50 years with a range between 16–101, so is not restricted to or concentrated on older adults which is a common limitation of existing loneliness research [16–19,22,28–30]. The majority of respondents in Understanding Society were white, which is representative of the UK population according to the 2021 Census (82% ‘white’ ethnic group [55] but excludes ethnic minorities, a gap recognised in a recent review which suggested particular need for quantitative analysis and consideration of intervention approaches in this population [56].

Around 40% of the sample had some experience of loneliness, of which around 32% experienced loneliness ‘some of the time’ and around 8% experienced loneliness ‘often’, being the most severe. This is consistent with recent figures from the Office for National Statistics who found that around a quarter of UK adults experience loneliness often, always or sometimes, of which only 7% felt lonely often or always [57]. The findings are also consistent with the BBC Loneliness Experiment which found 40% of 16–24-year-olds felt lonely often, compared to 27% of respondents aged over 75 [58]. The UCLA loneliness score is determined based on three dimensions: feeling lack of companionship, feeling left out, and feeling isolated from others. In this sample, slightly fewer respondents said they ‘often’ feel ‘left out’ (6%) than often lacked companionship (8%) and/or felt isolated from others (8%). This is consistent with the definition of loneliness that one can feel lonely even with lots of people around so not physically isolated [7].

A greater association is observed between loneliness and the health and wellbeing related outcomes when defining loneliness as experienced often than when including any experience of loneliness (often or sometimes) in the definition for analysis. Little difference is detected overall when comparing results for the UCLA loneliness scale to the single item question for loneliness. Further work is however required to consider the statistical significance of any difference. The need for deeper consideration of a single item question compared to a multi-item scale for loneliness has also been suggested as a result of COVID-19 given the evolving perceptions of loneliness and similar administrative protocols for both measurements [59]. Additionally, there could be benefits to considering the use of the different loneliness measurement methods in different populations, by for example age or ethnicity, or in populations where loneliness is perceived differently.

Loneliness is associated to lower physical functioning (SF-12 physical subscale), poorer mental health (SF-12 mental subscale), greater psychological distress (GHQ-12), and lower positive mental wellbeing (SWEMWBS). Amongst individuals experiencing loneliness, SF-12 scores fell within the threshold for signifying a physical condition and indicating clinical depression. Meanwhile, this threshold was not reached when considering the mean scores for the group not experiencing loneliness. This was the case when both including and excluding ‘sometimes lonely’ from the definition of loneliness and suggests a greater propensity for more serious physical and mental conditions when experiencing loneliness than when not currently experiencing loneliness. Furthermore, when restricting the definition of loneliness to something experienced ‘often’ the GHQ-12 and SWEMWBS indicate poor mental health and mild depression respectively amongst respondents experiencing loneliness, but not those considered not-lonely by this definition.

A greater number of GP visits were observed by individuals experiencing loneliness. Additionally, lonely individuals attended outpatient hospital or clinic visits more frequently and report a higher number of inpatient days than respondents not experiencing loneliness. When combining GP appointments, outpatient and inpatient visits, consistently higher NHS service costs are observed for individuals experiencing loneliness. When loneliness is defined as feeling lonely ‘often’, the difference in cost of these healthcare services, dependent on lower and upper bound assumptions, is predicted between £813.35 and £885.49 with a greater cost incurred by those experiencing loneliness. This difference in cost falls to between £352.39 and £409.42 when including ‘sometimes’ in the definition of an individual experiencing loneliness. However, it is important to note that GP and outpatient appointments are indicated in the data as categorical variables, thus difference in mean does not accurately reflect the difference in number of visits in its raw form. Consequently, median scores are representative of response option rather than number of visits.

The greatest contributor to total NHS service cost is inpatient bed days. One explanation for inpatient days acting as a cost driver could be the higher unit cost (£1,111). Of note, while this is the most accurate figure from PSSRU, the reason for inpatient admission is not reported in the data set, which could influence the associated cost of hospitalisation. These figures should therefore be considered with caution. Costs could also be driven by a more accurate upper bound of the number of inpatient days/visits as this is self-reported as a specific number rather than with pre-defined response options, and so does not necessitate what might be a conservative assumption of the maximum service use. Furthermore, multiple inpatient days may be experienced for the same episode contributing to a higher number of total days whereas GP and outpatient visits are contained to a single day/session.

The difference in healthcare costs associated to loneliness compared to no experience of loneliness increases with age. When considering a more severe experience of loneliness it is further suggested that the age-related cost of loneliness could be U-shaped, consistent with existing research on loneliness prevalence [6,60]. Findings suggest a more significant impact of loneliness on health and related costs in those aged 16–24. This extends existing research of the cost of loneliness which does not differentiate a specific young adult population [31]. It also suggests a need to concentrate policy in support of the health and wellbeing of younger people. Regarding older adults, while the cost difference between lonely and non-lonely individuals is large in magnitude, it is only statistically significant when using a wider definition of loneliness (experienced sometimes or often). We suggest this could be due to older people having higher health-related costs more generally for both groups, whereas younger people are generally healthier and so the difference in service use between lonely and non-lonely individuals is more significant.

Findings of a greater cost associated to NHS service use in lonely individuals is consistent with previous research which, while limited in quantity, scope, and demographics, predominantly reports excess healthcare costs associated to loneliness [25]. This also supports suggestions that interventions for loneliness could reduce avoidable healthcare costs, and public sector costs more widely [7,61].

## Strengths and limitations

This study considers data from a large nationally representative sample of UK adults aged 16 and over where healthcare is free at the point of need. An additional strength to this paper is that it considers health, wellbeing, quality of life and cost outcomes in the UK. The data provide insight into a wide variety of health-related outcomes across a large population providing good evidence of association to loneliness, however, as is common in secondary data analysis, definitive interpretation of causality is limited. Additionally, though this research adjusts for observed covariates, the exploration of specific mediators, such as depression, was beyond the scope of the study. Though this could be considered as important in future work, previous research suggests the relationship between loneliness and both quality of life and substance abuse may be independent of depression [23,62].

## Future research

Future research should consider the impact of loneliness on health-related quality of life and health service use in greater detail for younger (not older adult) populations and ethnic minorities as these remain under-studied populations. Additionally, there is some evidence of greater statistical significance between costs at age 50–75 years suggesting benefit to further research in this population. Finally, greater consideration of the wider economic outcomes related to loneliness, such as productivity (absenteeism and presenteeism), unemployment, and social capital should be evaluated in greater detail to provide a more complete picture of the economic cost of loneliness [2,3].

## Conclusion

Overall, loneliness is associated with poorer psychological, physical, and mental health outcomes as well as poorer wellbeing and quality of life. There is also evidence of greater NHS service use, and therefore higher resulting costs, amongst individuals experiencing loneliness, particularly youngest and oldest adults. Findings are consistent, comparing populations ‘often’ to ‘often or sometimes’ experiencing loneliness, with greater magnitude of association between loneliness and health economic related outcomes when restricting analysis to a definition that loneliness occurs when experienced ‘often’.

## Supporting information

S1 Table Unit costs for health care services. (PDF)

S2 Table Difference in mean (unadjusted average marginal effects). (PDF)

S3 Table Cost of health care service use based on unadjusted mean service use. (PDF)
