# Supplementary material for: How loneliness relates to health, wellbeing, quality of life, and healthcare resource utilisation and costs across multiple age groups in the UK
Source: PLoS One. 2025 Sep 3;20(9):e0327671. doi: 10.1371/journal.pone.0327671 (PMC12407476; doi:10.1371/journal.pone.0327671)
Supplement: S1 Table — (PDF) [file pone.0327671.s001.pdf]

# Supporting Information 1

## S1 Table. Unit costs for health care services

| Service | Unit cost (£) | Unit | Source | Page | Comment |
|---|---|---|---|---|---|
| GP at surgery | 49 | 10-minute consultation | PSSRU 2023 | 64 | Per surgery consultation lasting 10 minutes. With qualification costs including direct care staff costs |
| Outpatient appointment | 217 | Outpatient attendance | PSSRU 2023 | 36 | |
| Inpatient day case | 1,111 | Day case | PSSRU 2023 | 36 | |
