# Supplementary material for: How loneliness relates to health, wellbeing, quality of life, and healthcare resource utilisation and costs across multiple age groups in the UK
Source: PLoS One. 2025 Sep 3;20(9):e0327671. doi: 10.1371/journal.pone.0327671 (PMC12407476; doi:10.1371/journal.pone.0327671)
Supplement: S2 Table — (PDF) [file pone.0327671.s002.pdf]

Supporting Information 2

S2 Table. Difference in mean (unadjusted average marginal effects) ϕ

	UCLA						Direct question					
	Lonely = often lonely			Lonely = sometimes or often lonely			Lonely = often lonely			Lonely = sometimes or often lonely		
	Lonely N=1,385	Not lonely N=21,686	Difference N=23,071	Lonely N=9,475	Not lonely N=13,596	Difference N=23,071	Lonely N=1,898	Not lonely N=21,173	Difference N=23,071	Lonely N=9,252	Not lonely N=13,819	Difference N=23,071
**Wellbeing and Quality of Life**												
SF12 – PCS	46.87 (13.70) [5.90, 74.17]	49.98 (10.24) [5.98, 74.13]	-3.12*** (0.29) [-3.69,-2.55]	48.60 (11.83) [5.9, 74.17]	50.63 (9.37) [5.98, 72.81]	-2.04*** (0.14) [-2.31,-1.77]	47.43 (13.66) [9.68, 74.17]	50.01 (10.15) [5.90, 74.13]	-2.58*** (0.25) [-3.07,-2.08]	48.80 (11.81) [5.90, 74.17]	50.47 (9.47) [5.98, 72.81]	-1.67*** (0.14) [-1.95,-1.40]
SF12 - MCS	32.72 (11.88) [0.03, 64.89]	48.39 (10.22) [0, 74.46]	-15.67*** (0.29) [-16.23, -15.11]	41.18 (11.20) [0, 69.03]	51.82 (8.41) [5.14, 74.46]	-10.64*** (0.13) [-10.90, -10.39]	33.07 (11.91) [0, 68.13]	48.74 (9.92) [0, 74.46]	-15.67*** (0.24) [-16.14, -15.19]	40.97 (11.22) [0, 69.03]	51.79 (8.37) [5.14, 74.46]	-10.82*** (0.13) [-11.07, -10.56]
GHQ-12 (Likert)	19.94 (7.60) [1, 36]	11.19 (5.17) [0, 36]	8.75*** (0.21) [8.46, 9.04]	14.85 (6.38) [0, 36]	9.53 (3.98) [0, 36]	5.31*** (0.07) [5.18, 5.45]	19.71 (7.53) [1, 36]	11.00 (4.95) [0, 36]	8.71*** (0.12) [8.46, 8.95]	14.88 (6.40) [0, 36]	9.60 (4.03) [0, 36]	5.28*** (0.07) [5.14, 5.41]
GHQ-12 (Caseness)	6.34 (4.23) [0, 12]	1.67 (2.92) [0, 12]	4.67*** (0.08) [4.51, 4.84]	3.47 (3.91) [0, 12]	0.89 (2.02) [0, 12]	2.58*** (0.04) [2.51, 2.66]	6.26 (4.24) [0, 12]	1.56 (2.80) [0, 12]	4.70*** (0.07) [4.56, 4.84]	3.47 (3.94) [0, 12]	0.93 (2.07) [0, 12]	2.54*** (0.04) [2.46, 2.62]
sWEMWBS	18.01 (4.77) [7, 35]	24.74 (4.56) [7, 35]	-6.73*** (0.13) [-6.98,-6.48]	21.47 (4.31) [7, 35]	26.34 (4.13) [7, 35]	-4.87*** (0.06) [-4.98,-4.76]	18.38 (4.62) [7, 35]	24.87 (4.49) [7, 35]	-6.49*** (0.11) [-6.70,-6.28]	21.44 (4.29) [7, 35]	26.27 (4.18) [7, 35]	-4.83*** (0.06) [-4.94,-4.72]
**Health care service use**												
Visited GP in last 12 months	1.28 (1.19) [0, 4] {1}	0.87 (0.95) [0, 4] {1}	0.41*** (0.03) [0.35, 0.46]	1.04 (1.06) [0, 4] {1}	0.79 (0.88) [0, 4] {1}	0.25*** (0.01) [0.23, 0.28]	1.27 (1.19) [0, 4] {1}	0.86 (0.94) [0, 4] {1}	0.41*** (0.02) [0.36, 0.45]	1.05 (1.06) [0, 4] {1}	0.79 (0.89) [0, 4] {1}	0.25*** (0.01) [0.23, 0.28]
Hospital or clinic out-patient last 12 months	0.70 (1.02) [0, 4] {0}	0.51 (0.84) [0, 4] {0}	0.19*** (0.02) [0.14, 0.23]	0.60 (0.92) [0, 4] {0}	0.47 (0.80) [0, 4] {0}	0.13*** (0.01) [0.10, 0.15]	0.70 (1.02) [0, 4] {0}	0.51 (0.84) [0, 4] {0}	0.19*** (0.02) [0.15, 0.23]	0.61 (0.93) [0, 4] {0}	0.47 (0.80) [0, 4] {0}	0.13*** (0.01) [0.11, 0.15]
Hospital or clinic in-patient last 12 months	0.10 (0.30) [0, 1] {0}	0.06 (0.24) [0, 1] {0}	0.04*** (0.01) [0.03, 0.06]	0.08 (0.26) [0, 1] {0}	0.06 (0.23) [0, 1] {0}	0.02*** (0.00) [0.01, 0.03]	0.10 (0.30) [0, 1] {0}	0.06 (0.24) [0, 1] {0}	0.04*** (0.01) [0.03, 0.05]	0.08 (0.27) [0, 1] {0}	0.06 (0.23) [0, 1] {0}	0.02*** (0.00) [0.01, 0.03]
In-patient number of days	1.31 (8.94) [0, 200] {0}	0.45 (3.98) [0, 180] {0}	0.86*** (0.12) [0.62, 1.10]	0.71 (5.73) [0, 200] {0}	0.36 (3.25) [0, 180] {0}	0.35*** (0.06) [0.24, 0.47]	1.10 (7.63) [0, 200] {0}	0.45 (4.03) [0, 180] {0}	0.65*** (0.11) [0.44, 0.86]	0.71 (5.71) [0, 200] {0}	0.37 (3.33) [0, 180] {0}	0.34*** (0.06) [0.22, 0.46]

ϕ Mean (SD) [range] {median}; Difference = Mean (SE) [95%CI]
