# Supplementary material for: How loneliness relates to health, wellbeing, quality of life, and healthcare resource utilisation and costs across multiple age groups in the UK
Source: PLoS One. 2025 Sep 3;20(9):e0327671. doi: 10.1371/journal.pone.0327671 (PMC12407476; doi:10.1371/journal.pone.0327671)
Supplement: S3 Table — (PDF) [file pone.0327671.s003.pdf]

Supporting Information 3

S3 Table. Cost of health care service use based on unadjusted mean service use $^{\phi}$

Health care service (use in last 12 months)	Unit cost	UCLA						Direct question					
		Lonely = often lonely			Lonely = sometimes or often lonely			Lonely = often lonely			Lonely = sometimes or often lonely		
		**Lonely** N=1,385	**Not lonely** N=21,686	**Difference** N=23,071	**Lonely** N=9,475	**Not lonely** N=13,596	**Difference** N=23,071	**Lonely** N=1,898	**Not lonely** N=21,173	**Difference** N=23,071	**Lonely** N=9,252	**Not lonely** N=13,819	**Difference** N=23,071
**Mean service use (unadjusted)**													
GP visit *[lower bound]*	n/a	2.23<br>(2.97)<br>[0, 11]<br>{1}	1.28<br>(2.00)<br>[0, 11]<br>{1}	0.95***<br>(0.06)<br>[0.84, 1.06]	1.66<br>(2.43)<br>[0, 11]<br>{1}	1.11<br>(1.76)<br>[0, 11]<br>{1}	0.55***<br>(0.03)<br>[0.50, 0.61]	2.21<br>(2.96)<br>[0, 11]<br>{1}	1.26<br>(1.97)<br>[0, 11]<br>{1}	0.96***<br>(0.05)<br>[0.86, 1.05]	1.67<br>(2.43)<br>[0, 11]<br>{1}	1.12<br>(1.78)<br>[0, 11]<br>{1}	0.55***<br>(0.03)<br>[0.49, 0.60]
GP visit *[upper bound]*	n/a	4.29<br>(6.34)<br>[0, 25]<br>{2}	2.40<br>(4.03)<br>[0, 25]<br>{2}	1.89***<br>(0.12)<br>[1.67, 2.12]	3.15<br>(5.05)<br>[0, 25]<br>{2}	2.07<br>(3.47)<br>[0, 25]<br>{2}	1.08***<br>(0.06)<br>[0.97, 1.19]	4.26<br>(6.32)<br>[0, 25]<br>{2}	2.35<br>(3.95)<br>[0, 25]<br>{2}	1.91***<br>(0.10)<br>[1.71, 2.10]	3.15<br>(5.05)<br>[0, 25]<br>{2}	2.08<br>(3.51)<br>[0, 25]<br>{2}	1.07***<br>(0.06)<br>[0.96, 1.18]
Hospital or clinic out-patient visit *[lower bound]*	n/a	1.15<br>(2.31)<br>[0, 11]<br>{0}	0.76<br>(1.71)<br>[0, 11]<br>{0}	0.39***<br>(0.05)<br>[0.30, 0.49]	0.93<br>(1.96)<br>[0, 11]<br>{0}	0.69<br>(1.60)<br>[0, 11]<br>{0}	0.24***<br>(0.02)<br>[0.19, 0.29]	1.15<br>(2.29)<br>[0, 11]<br>{0}	0.75<br>(1.70)<br>[0, 11]<br>{0}	0.39***<br>(0.04)<br>[0.31, 0.48]	0.94<br>(1.99)<br>[0, 11]<br>{0}	0.68<br>(1.57)<br>[0, 11]<br>{0}	0.26***<br>(0.02)<br>[0.22, 0.31]
Hospital or clinic out-patient visit *[upper bound]*	n/a	1.82<br>(3.11)<br>[0, 13]<br>{0}	1.27<br>(2.45)<br>[0, 13]<br>{0}	0.55***<br>(0.07)<br>[0.42, 0.69]	1.52<br>(2.74)<br>[0, 13]<br>{0}	1.16<br>(2.30)<br>[0, 13]<br>{0}	0.36***<br>(0.03)<br>[0.30, 0.43]	1.82<br>(3.10)<br>[0, 13]<br>{0}	1.26<br>(2.43)<br>[0, 13]<br>{0}	0.56***<br>(0.06)<br>[0.44, 0.68]	1.53<br>(2.78)<br>[0, 13]<br>{0}	1.15<br>(2.28)<br>[0, 13]<br>{0}	0.38***<br>(0.03)<br>[0.32, 0.45]
Hospital or clinic in-patient (number of days)	n/a	1.31<br>(8.94)<br>[0, 200]<br>{0}	0.45<br>(3.98)<br>[0, 180]<br>{0}	0.86***<br>(0.12)<br>[0.62, 1.10]	0.71<br>(5.73)<br>[0, 200]<br>{0}	0.36<br>(3.25)<br>[0, 180]<br>{0}	0.35***<br>(0.06)<br>[0.24, 0.47]	1.10<br>(7.63)<br>[0, 200]<br>{0}	0.45<br>(4.03)<br>[0, 180]<br>{0}	0.65***<br>(0.11)<br>[0.44, 0.86]	0.71<br>(5.71)<br>[0, 200]<br>{0}	0.37<br>(3.33)<br>[0, 180]<br>{0}	0.34***<br>(0.06)<br>[0.22, 0.46]
**Cost of mean service use (£)**													
GP visit *[lower bound]*	£49	109.39<br>(145.42)<br>[0, 539]<br>{49}	62.77<br>(97.92)<br>[0, 539]<br>{49}	46.62***<br>(2.81)<br>[41.12, 52.13]	81.54<br>(119.25)<br>[0, 539]<br>{49}	54.43<br>(86.27)<br>[0, 539]<br>{49}	27.11***<br>(1.35)<br>[24.46, 29.77]	108.53<br>(144.96)<br>[0, 539]<br>{49}	61.71<br>(96.30)<br>[0, 539]<br>{49}	46.82***<br>(2.42)<br>[42.07, 51.57]	81.67<br>(119.18)<br>[0, 539]<br>{49}	54.78<br>(87.00)<br>[0, 539]<br>{49}	26.89***<br>(1.36)<br>[24.23, 29.55]
GP visit *[upper bound]*	£49	210.26<br>(310.43)<br>[0, 1225]<br>{98}	117.44<br>(197.42)<br>[0, 1225]<br>{98}	92.82***<br>(5.71)<br>[81.63, 104.01]	154.25<br>(247.66)<br>[0, 1225]<br>{98}	101.24<br>(170.01)<br>[0, 1225]<br>{98}	53.01***<br>(2.75)<br>[47.62, 58.40]	208.81<br>(309.86)<br>[0, 1225]<br>{98}	115.32<br>(193.46)<br>[0, 1225]<br>{98}	93.49***<br>(4.92)<br>[83.83, 103.14]	154.44<br>(247.47)<br>[0, 1225]<br>{98}	101.96<br>(171.82)<br>[0, 1225]<br>{98}	52.48***<br>(2.76)<br>[47.07, 57.89]
Hospital or clinic out-patient visit *[lower bound]*	£217	249.90<br>(500.42)<br>[0, 2387]<br>{0}	165.11<br>(371.97)<br>[0, 2387]<br>{0}	84.80***<br>(10.56)<br>[64.10, 105.49]	200.90<br>(424.39)<br>[0, 2387]<br>{0}	148.80<br>(346.77)<br>[0, 2387]<br>{0}	52.10***<br>(5.09)<br>[42.12, 62.08]	248.78<br>(496.31)<br>[0, 2387]<br>{0}	163.15<br>(368.58)<br>[0, 2387]<br>{0}	85.63***<br>(9.12)<br>[67.75, 103.51]	204.17<br>(432.17)<br>[0, 2387]<br>{0}	147.45<br>(341.41)<br>[0, 2387]<br>{0}	56.72***<br>(5.11)<br>[46.70, 66.74]

Hospital or clinic out-patient visit *[upper bound]*	£217	395.46 (674.52) [0, 2821] {0}	275.66 (531.86) [0, 2821] {0}	119.80*** (15.01) [90.38, 149.21]	329.04 (595.25) [0, 2821] {0}	250.66 (499.45) [0, 2821] {0}	78.38*** (7.24) [64.19, 92.56]	394.67 (673.61) [0, 2821] {0}	272.83 (527.72) [0, 2821] {0}	121.84**** (12.97) [96.43, 147.26]	332.70 (602.62) [0, 2821] {0}	249.47 (494.92) [0, 2821] {0}	83.23*** (7.26) [68.99, 97.46]
Hospital or clinic in-patient (number of days)	£1,111	1457.54 (9937.59) [0, 222200] {0}	503.50 (4422.94) [0, 199980] {0}	954.04*** (136.66) [686.17, 1221.90]	792.06 (6362.37) [0, 222200] {0}	399.59 (3615.48) [0, 199980] {0}	392.48*** (66.01) [263.10, 521.85]	1222.80 (8474.72) [0, 222200] {0}	501.43 (4479.96) [0, 199980] {0}	721.38*** (118.18) [489.74, 953.01]	787.98 (6345.89) [0, 222200] {0}	408.66 (3695.78) [0, 199980] {0}	379.32*** (66.26) [249.45, 509.20]
**Total health service cost** *[lower bound]*	n/a	**1816.83** (10075.33) [0, 222347] {147}	**731.37** (4533.66) [0, 200736] {49}	**1085.46***** (139.71) [811.61, 1359.30]	**1074.51** (6488.50) [0, 222347] {49}	**602.82** (3715.11) [0, 199980] {49}	**471.69***** (67.48) [339.43, 603.95]	**1580.12** (8622.70) [0, 222347] {147}	**726.29** (4586.78) [0, 200736] {49}	**853.83***** (120.81) [617.03, 1090.62]	**1073.82** (6472.54) [0, 222347] {49}	**610.89** (3794.88) [0, 199980] {49}	**462.93***** (67.74) [330.16, 595.70]
**Total health service cost** *[upper bound]*	n/a	**2063.25** (10148.68) [0, 222445] {245}	**896.59** (4603.04) [0, 201639] {98}	**1166.66***** (141.58) [889.15, 1444.16]	**1275.35** (6564.81) [0, 222445] {98}	**751.48** (3777.05) [0, 199980] {98}	**523.87***** (68.38) [389.84, 657.89]	**1826.28** (8705.33) [0, 222445] {245}	**889.57** (4653.55) [0, 201639] {98}	**936.71***** (122.42) [696.75, 1176.67]	**1275.12** (6547.82) [0, 222445] {98}	**760.09** (3858.11) [0, 199980] {98}	**515.03***** (68.64) [380.49, 649.56]

Lower (upper) bound = estimated based on lower (upper) bound of response options in the range.

ϕ Mean (SD) [range] {median}; Difference = Mean (SE) [95%CI]

*p<0.05, **p<0.01, ***p<0.001
